# Outcomes of pathologic stage T3a renal cell carcinoma up-staged from small renal tumor: emphasis on partial nephrectomy

**DOI:** 10.1186/s12885-018-4338-1

**Published:** 2018-04-16

**Authors:** Hakmin Lee, Minseung Lee, Sang Eun Lee, Seok-Soo Byun, Hyeon Hoe Kim, Cheol Kwak, Sung Kyu Hong

**Affiliations:** 1 Department of Urology, Seoul National University Bundang Hospital, 82, Gumi-ro 173 Beon-gil, Bundang-gu, Seongnam-si, Gyeonggi-do 13620 South Korea; 2 Department of Urology, Seoul National University Hospital, Seoul, South Korea; 3 Department of Urology, Seoul National University College of Medicine, Seoul, South Korea

**Keywords:** Upstaging, Renal cell carcinoma, Nephrectomy, Survival, Stage

## Abstract

**Background:**

The prognosis of patients with pathologic stage T3a renal cell carcinoma (RCC) that is up-staged from a small renal tumor remains controversial. We evaluated the prognosis of patients with RCC who were up-staged from clinical stage T1 to pathologic stage T3a.

**Methods:**

We retrospectively reviewed the data of 3431 patients who were surgically treated for clinical stage T1 RCC. The survival outcomes were compared using Kaplan-Meier and Cox proportional analyses.

**Results:**

Among the clinical stage T1 patients, 215 (6.3%) were finally up-staged to pathologic stage T3a. Patient age (HR 1.302, 95% CI 1.018–1.046, *p* <  0.001), tumor diameter (HR 1.686, 95% CI 1.551–1.834, *p* <  0.001), and hilar location (HR 1.765, 95% CI 1.147–2.715, *p* = 0.010) were significantly associated with upstaging. Kaplan-Meier analyses showed significantly shorter recurrence-free, cancer-specific and overall survivals (all *p* <  0.001) in patients who were up-staged. Multivariate Cox analyses revealed pathologic upstaging as an independent predictor of shorter recurrence-free (HR 2.195, 95% CI 1.459–3.300, *p* <  0.001), cancer-specific (HR 2.238, 95% CI 1.252–4.003, *p* = 0.007), and overall survivals (HR 1.632, 95% CI 1.029–2.588, *p* = 0.037). Subgroup analysis of pathologic stage T3a showed no significant difference in survival of the partial nephrectomy group when compared to the radical nephrectomy group (all *p* > 0.5).

**Conclusions:**

Patients up-staged from clinical stage T1 to pathologic stage T3a RCC showed shorter survival outcomes than those without upstaging. However, partial nephrectomy, compared with radical nephrectomy, showed comparable outcomes in patients who were up-staged.

## Background

Renal cell carcinoma (RCC) is the most frequently diagnosed malignancy among renal tumors worldwide [1], and its incidence continues to steadily increase in most countries [2]. The advancements and penetration of modern radiologic imaging tools, including computed tomography (CT) and ultrasonography, have contributed to the overall increase in the incidental detection of RCC, particularly that of the localized disease [3]. A recent, large epidemiologic study from the United States showed that patients with loco-regional disease comprised over 80% of the RCC patient population [4]. Another study using the Surveillance, Epidemiology and End Results (SEER) database also demonstrated that there was a concomitant increase in asymptomatic small RCC along with the overall increase in the incidence of RCC [5]. The major guidelines of both the European Association of Urology and the American Urologic Association recommend partial nephrectomy as the primary treatment for clinical stage T1 RCC to facilitate better preservation of renal function and equivalent oncological control [6, 7].

However, there have been concerns regarding patients who were initially diagnosed with a clinical stage T1 renal tumor that was subsequently up-staged to a higher pathologic stage, particularly following partial nephrectomy. Previous studies have reported heterogeneous oncological outcomes between patients who experienced pathologic upstaging (clinical stage T1 to pathologic stage T3a) and those who did not [8–11]. Some reported similar survival outcomes between the two groups, while others showed contradictory results. Another important issue in this regard is the lack of unified application of the recent TNM classification. Most previous studies used the previous definition of TNM staging and/or did not provide exact definitions for the pathologic stage T3a. Therefore, we tried to evaluate the oncological outcomes and exact prognosis of patients with clinical stage T1 RCC in our cohort whose cancers were upstaged from clinical stage T1 to pathologic stage T3a after surgery using the recent definitions of the 2010 TNM classification.

## Methods

After approval from the Institutional Ethical Review Board, we retrospectively reviewed the medical records of 3749 patients who underwent surgery between January 1997 and December 2016 for clinical stage T1 renal tumors and who were diagnosed with RCC without any evidence of metastasis. Renal biopsy was not routinely performed even if the patients were scheduled to undergo partial nephrectomy. If there were any suspicious findings indicative of an advanced clinical stage, such as venous invasion or thrombus, patients were regarded as having disease higher than clinical stage T1, and 203 patients were subsequently excluded from the study. After the additional exclusion of 115 patients (other malignancy [*n* = 57], lymph node invasion [*n* = 12], and incomplete information [*n* = 46]), we finally included 3431 patients. The clinical and pathologic information was retrieved from our prospectively maintained database. The clinical stages were mainly determined using preoperative abdominal CT. The preoperative evaluation also included chest radiography (or CT) and a bone scan. If needed, further evaluations, such as magnetic resonance imaging or ultrasonography, were also performed. The clinical and pathologic stages were determined according to the seventh edition of the TNM classification of the American Joint Committee on Cancer [12]. We centrally reviewed the medical records of patients who were initially staged according to the outdated TNM staging and reclassified them according to the recent 2010 TNM classification. Nuclear grading was performed using the Fuhrman’s grading system and histologic subtypes were assessed using the Heidelberg classification [13, 14]. Pathologic upstaging was defined when the final pathology was determined as pathologic stage T3a for clinical stage T1 renal tumors. When the tumor was located in the renal hilum in close contact with the renal vessels (≤0.5 cm), it was categorized as a hilar tumor. We also classified the patients into three groups according to the depth of the tumor, as in previous studies [15]. An exophytic tumor was defined when ≥60% of the tumor protruded externally from the parenchymal surface, an endophytic tumor was defined when ≥60% of the tumor was embedded inside the parenchyma, and all other tumors were defined as mesophytic tumors. Disease recurrence was defined when there was radiologic or pathologic evidence of local recurrence and/or distant metastasis. Postoperative evaluations were performed at 3- to 6-month intervals for the first 2 years and annually thereafter. Information about patient mortality was acquired from the database of the Korean National Statistical Office and by review of our medical records. Recurrence-free survival, cancer-specific survival, and overall survival were defined as the time from the date of surgery to the date of recurrence, cancer-specific mortality, or all-cause mortality, respectively. We performed subgroup analyses to compare the oncological outcomes between partial and radical nephrectomy in the clinical stage T1a patients and in the clinical stage T1 patients with pathologic upstaging.

An independent *t*-test and chi-square test were performed to compare the perioperative characteristics between the groups. Logistic regression tests were performed for univariate and multivariate analyses and Kaplan–Meier analysis with log-rank tests to evaluate the differences in survival outcomes between the subgroups. Multivariate Cox-proportional hazard analysis was performed to identify the possible predictors of each survival outcome. The SPSS software package (SPSS 19.0, Chicago, IL, USA) was used for the statistical analyses. All *p*-values presented were two-sided and *p* <  0.05 was considered statistically significant.

## Results

The patients’ clinico-pathologic profiles are summarized in Table 1. The median age was 55.0 years (interquartile range (IQR) 46.0–65.0), the median tumor size was 3.3 cm (IQR 2.0–4.4), and the median follow-up time was 39.0 months (IQR 15.0–69.0). There were 210 (6.1%) patients with hilar tumors, 944 (28%) patients with endophytic tumors, 809 patients (24%) with mesophytic tumors, and 1678 (49%) patients with exophytic tumors. Partial nephrectomy was performed in 2071 (60%) patients and radical nephrectomy in 1360 (40%) patients. After partial or radical nephrectomy, 215 (6.3%) patients showed pathologic upstaging and no patients were treated with adjuvant therapy for pathologic upstaging. Comparison of the clinical characteristics of the two groups revealed that patients with pathologic upstaging were significantly older (*p* <  0.001), had significantly larger tumors (*p* <  0.001), had more co-morbidities, such as diabetes mellitus (*p* = 0.028) or hypertension (*p* = 0.001), and had a significantly higher prevalence of hilar tumors (*p* <  0.001). Forty-seven (1.4%) patients (five in the radical nephrectomy group and 42 in the partial nephrectomy group) showed positive surgical margins and four were upstaged to T3a (one in the radical nephrectomy group and three in the partial nephrectomy group). Although 11 patients were initially intended to undergo partial nephrectomy, they were inevitably converted to radical nephrectomy. The patients with pathologic upstaging also showed a significantly higher nuclear grade than those without pathologic upstaging (*p* <  0.001). The multivariate regression tests showed that older age, larger tumor size, and hilar location were significantly associated with pathologic upstaging, whereas tumor depth did not show any significant associations (Table 2).

**Table 1:** Summarization of clinical and pathologic characteristics of entire patients and according to the up-staging of pathologic stages after surgical treatments for clinical stage T1 renal cell carcinoma

	Entire patients (*n* = 3431)	Patients without up-staging (*n* = 3216)	Patients with up-staging (*n* = 215)	*p* value
Preoperative characteristics				
Median Age (y)	55.0 (46.0–65.0)	55.0 (46.0–65.0)	60 (52.0–69.0)	< 0.001
Median BMI (*kg*/*m*^2^)	24.6 (22.6–26.7)	24.6 (22.6–26.7)	24.4 (22.7–26.7)	0.406
Gender (male)	2462 (72%)	2304 (72%)	158 (74%)	0.585
ECOG score (≥1)	802 (23%)	749 (23%)	53 (25%)	0.677
Diabetes mellitus	502 (15%)	459 (14%)	43 (20%)	0.028
Hypertension	1351 (40%)	1244 (39%)	107 (50%)	0.001
Tumour size (cm)	3.3 (2.0–4.4)	3.1 (1.9–4.1)	5.0 (3.7–6.2)	< 0.001
Laparoscopy	1420 (41%)	1355 (42%)	85 (40%)	0.617
Type of nephrectomy				< 0.001
Radical	1360 (40%)	1202 (37%)	158 (74%)	
Partial	2071 (60%)	2014 (63%)	57 (27%)	
Hilar location	210 (6.1%)	174 (5.4%)	36 (17%)	< 0.001
Tumour location				0.204
Exophytic	1678 (49%)	1565 (49%)	113 (53%)	
Mesophytic	809 (24%)	769 (24%)	40 (19%)	
Endophytic	944 (28%)	882 (27%)	62 (29%)	
Clinical stages				< 0.001
cT1a	2462 (72%)	2379 (74%)	83 (39%)	
cT1b	969 (28%)	837 (26%)	132 (61%)	
Postoperative characteristics				
Pathologic stage				< 0.001
pT1	2406 (70%)	2406 (75%)		
pT2	810 (24%)	810 (25%)		
pT3a	215 (6.3%)		215 (100%)	
Fuhrman grade				< 0.001
≤2	1935 (56%)	1865 (58%)	70 (33%)	
≥3	1496 (44%)	1351 (42%)	145 (67%)	
Histologic subtype				0.234
Clear cell	2912 (85%)	2737 (85%)	175 (81%)	
Papillary	238 (6.9%)	223 (6.9%)	15 (7.0%)	
Chromophobe	236 (6.9%)	217 (6.7%)	19 (8.8%)	
Collecting duct	6 (0.2%)	5 (0.2%)	1 (0.5%)	
Unclassified	39 (1.1%)	34 (1.1%)	5 (0.1%)	

*BMI* body mass index, *ECOG* Eastern Cooperative Oncology Group

**Table 2:** Multivariate regression tests upon up-staging to pathologic stage T3a in 3431 patients surgically treated for localized renal cell carcinoma

	Adjusted with hilar location			Adjusted with tumour shape		
	HR	95% CI	*p* value	HR	95% CI	*p* value
Age	1.032	1.018–1.046	< 0.001	1.032	1.017–1.046	< 0.001
High BMI (≥24 *kg*/*m*^2^)	0.871	0.641–1.184	0.379	0.870	0.640–1.182	0.373
Gender (Female)	0.766	0.543–1.079	0.127	0.767	0.544–1.081	0.129
Diabetes mellitus (yes)	1.313	0.890–1.939	0.170	1.316	0.891–1.942	0.167
Hypertension (yes)	1.127	0.811–1.567	0.476	1.128	0.812–1.568	0.472
ECOG score (≥2)	0.567	0.238–1.355	0.202	0.575	0.241–1.376	0.214
Tumour size	1.686	1.551–1.834	< 0.001	1.726	1.588–1.877	< 0.001
Hilar location (yes)	1.765	1.147–2.715	0.010			
Tumour location						
Exophytic					Reference	
Mesophytic				0.805	0.539–1.202	0.288
Endophytic				1.318	0.925–1.879	0.126

*HR* hazard ratio, *CI* confidence interval, *BMI* body mass index, *ECOG* Eastern Cooperative Oncology Group

**Fig. 1:**
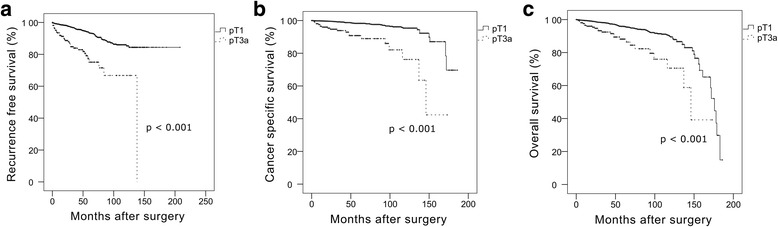
Kaplan-Meier analysis for recurrence-free, cancer-specific, and overall survival between patients with and without pathologic upstaging among the 3431 patients with clinical stage T1 renal cell carcinoma (**a**, recurrence-free survival; **b**, cancer-specific survival; **c**, overall survival)

**Fig. 2:**
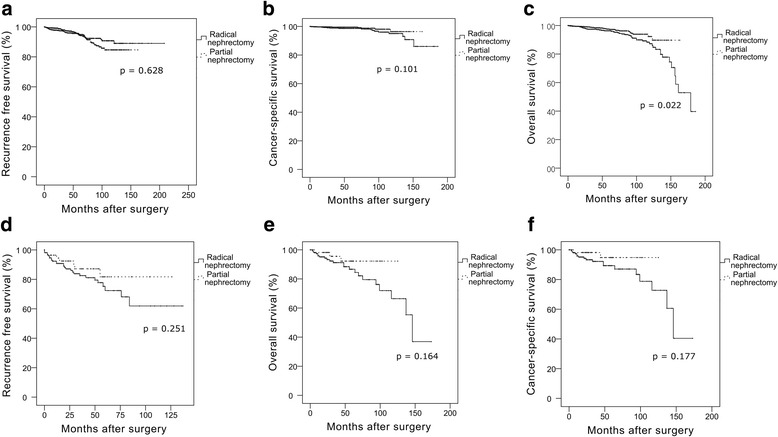
Kaplan-Meier analysis for recurrence-free, cancer-specific, and overall survivals between the type of surgery in patients with clinical stage T1a renal cell carcinoma (**a**–**c**) and in the patients with upstaging to pathologic stage T3a renal cell carcinoma (**d**–**f**)

After a median of 32.0 months (IQR 12.0–59.8), 196 (5.7%) patients showed disease recurrence. Among these 196 patients, 26 (13%) exhibited local recurrence abutting the resection margin or renal fossa, 169 (86%) presented with distant metastasis (with or without local recurrence), and one patient lacked information. Seventy-three cancer-specific mortalities occurred after a median of 43.0 months (IQR 18.5–92.5), and 156 overall mortalities occurred after a median of 50.0 months (IQR 22.3–91.8) postoperatively. Patients with pathologic upstaging also showed significantly shorter recurrence-free (*p* < 0.001), cancer-specific (*p* <  0.001), and overall survivals (*p* < 0.001) (Fig. 1). Among patients with pathologic upstaging, 36 showed disease recurrence (local recurrence, 4; distant metastasis, 32) after a median of 19.0 months (IQR 5.0–39.5), and 20 patients died from metastasis after a median of 28.5 months (IQR 9.5–86.5). The percentage of patients who had distant metastasis was significantly higher in patients with pathologic upstaging compared to patients without pathologic upstaging (*p* <  0.001). Multivariate Cox proportional analyses revealed pathologic upstaging as an independent predictor of shorter recurrence-free (HR 2.195, 95% CI 1.459–3.300, *p* <  0.001), cancer-specific (HR 2.238, 95% CI 1.252–4.003, *p* = 0.007), and overall survival (HR 1.632, 95% CI 1.029–2.588, *p* = 0.037) (Table 3). We performed subgroup analyses in only 2462 patients with clinical stage T1a RCC to compare the outcomes of radical nephrectomy with those of partial nephrectomy. Following Kaplan-Meier analyses, the partial nephrectomy group (*n* = 1782) showed equivalent oncological outcomes in terms of recurrence-free (*p* = 0.628) and cancer-specific survival (*p* = 0.101) compared with the radical nephrectomy group (*n* = 679) (Fig. 2). Moreover, patients who underwent partial nephrectomy showed significantly longer overall survival compared with patients who underwent radical nephrectomy (*p* = 0.022). Subsequently, we performed another subgroup analysis in the cohort of pathologic stage T3a patients (*n* = 215) who initially had clinical stage T1 RCC and upstaged to pathologic stage T3a; however, we could not find any significant differences in recurrence-free, cancer-specific, or overall survivals between the radical and partial nephrectomy groups (all *p* > 0.05, Fig. 2).

**Table 3:** Multivariate analysis using the Cox proportional hazard model of possible predictors of recurrence-free, overall and cancer-specific survivals after surgical treatments for localized renal cell carcinoma

	Recurrence-free survival			Overall survival			Cancer-specific survival		
	HR	95% CI	*p* value	HR	95% CI	*p* value	HR	95% CI	*p* value
Age	1.010	0.996–1.023	0.153	1.067	1.049–1.085	< 0.001	1.052	1.027–1.077	< 0.001
BMI (≥24 *kg*/*m*^2^)	0.834	0.623–1.115	0.219	0.682	0.491–0.948	0.023	0.732	0.451–1.189	0.207
Gender (Female)	0.832	0.592–1.168	0.288	0.834	0.574–1.210	0.339	0.842	0.494–1.437	0.529
Diabetes mellitus (yes)	0.930	0.599–1.443	0.746	1.797	1.244–2.595	0.002	2.124	1.251–3.606	0.005
Hypertension (yes)	1.094	0.792–1.510	0.586	1.022	0.717–1.457	0.903	1.110	0.651–1.894	0.701
ECOG score (≥1)	2.024	1.114–3.678	0.021	2.031	1.456–2.835	< 0.001	1.029	0.368–2.877	0.956
Tumour size	1.298	1.206–1.397	< 0.001	1.256	1.150–1.372	< 0.001	1.461	1.305–1.635	< 0.001
Fuhrman grade (≥3)	1.557	1.160–2.089	0.003	0.841	0.606–1.167	0.299	1.128	0.699–1.822	0.621
Pathologic up-staging to T3a	2.195	1.459–3.300	< 0.001	1.632	1.029–2.588	0.037	2.238	1.252–4.003	0.007
Cellular type (non-clear cell)	0.720	0.463–1.119	0.144	1.120	0.699–1.795	0.638	1.009	0.494–2.059	0.981

*HR* hazard ratio, *CI* confidence interval, *BMI* body mass index, *ECOG* Eastern Cooperative Oncology Group

## Discussion

In the present study, approximately 6.3% among the patients with clinical stage T1 RCC were upstaged to pathologic stage T3a after surgery. Furthermore, 17% of these patients with pathologic upstaging experienced disease recurrence, and 9.3% eventually died from the disease. Even though the percentage of patients who experienced pathologic upstaging after surgery was relatively low, a non-negligible percentage of these patients showed adverse consequences. Finally, the patients with pathologic upstaging showed significantly shorter survival outcomes in terms of recurrence-free, cancer-specific and overall survivals.

Previous studies in this field demonstrated conflicting results. Roberts et al. retrospectively analyzed 186 clinical stage T1 patients who underwent surgery at the Johns Hopkins Hospital, and concluded that patients with pathologic upstaging showed similar recurrence-free survival compared to those without pathologic upstaging [8]. However, their study utilized the 1997 TNM staging system and pathologic stage T3a was only defined when there were invasions of the adrenal gland and/or peri-renal fat. Another study by Ramaswamy et al. reported similar results after analyzing 494 patients with clinical stage T1 RCC after median follow-up time of 50 months [9]. They concluded that no disease recurrence occurred in the patients with pathologic upstaging, and therefore, pathologic upstaging was not associated with compromised oncologic outcomes. In contrast, Gorin et al. analyzed 563 patients with clinical stage T1 RCC who underwent robotic partial nephrectomy; they reported that pathologic stage T3a was associated with significantly shorter recurrence-free survival [10]. However, these previous studies had limitations, such as relatively small numbers of subjects and short follow-up periods.

The incidence of pathologic upstaging was also reported to be variable. Gorin et al. reported the incidence of pathologic upstaging as 4% of all clinical stage T1 patients [10], Ramaswamy et al. as 13% [9], and Roberts et al. as 31% [8]. Recently, Nayak et al. analyzed a large database of 1448 patients with clinical stage T1 RCC and reported that pathologic upstaging was observed in 134 (9%) patients [11]. In the present study, the overall incidence of pathologic upstaging in patients with clinical stage T1 RCC was 6.3%, which is similar to other studies’ results. As these incidences of pathologic upstaging are not high, our study clearly showed that patients with pathologic upstaging have worse clinical outcomes than those without pathologic upstaging. As partial nephrectomy is the first treatment option in patients with clinical T1a RCC, it is reasonable to question the oncologic feasibility of partial nephrectomy given that patients with pathologic upstaging have significantly worse clinical outcomes than those without pathologic upstaging. However, when we compared the oncologic outcomes between partial and radical nephrectomy in the patients with clinical stage T1a RCC, we could not find any significant differences in recurrence-free and cancer-specific survivals, suggesting that partial nephrectomy can provide at least equivocal oncological outcomes in patients with clinical T1a RCC, even with pathologic upstaging. Therefore, since our results showed that the incidence of pathologic upstaging was quite low, the majority of patients will still benefit from partial nephrectomy. Considering that the main reason for partial nephrectomy is the preservation of renal function and ultimately an increase in survival, we also compared overall survival between radical and partial nephrectomy. The partial nephrectomy group showed significantly longer overall survival compared with the radical nephrectomy group (*p* = 0.022). Furthermore, the subsequent subgroup analyses performed only with up-staged T3a patients showed no significant differences in each survival outcome (all *p* > 0.5). Other studies also previously reported increased survival benefits of partial nephrectomy in small RCC [16–19]. Thompson et al. analyzed 648 patients and concluded that overall survival was significantly shorter in the radical nephrectomy group [16]. Weight et al. performed a retrospective study of 2608 patients with clinical stage T1 renal tumors, and found significant survival advantages, along with an improved preservation of postoperative renal function in the partial nephrectomy group [17]. To the best of our knowledge, the present study is the largest study of the survival benefits of partial nephrectomy over radical nephrectomy in patients with clinical stage T1 RCC.

When we evaluated the tumor morphologic characteristics upon the occurrence of pathologic upstaging, we found that the hilar location of the tumor was significantly associated with an increased incidence of pathologic upstaging, whereas the depth of the tumor was not. To date, a small number of studies have sought to identify the preoperative risk factors associated with pathologic upstaging in patients with clinical stage T1 RCC [8–11]; however, only a few of these studies have tried to evaluate the impact of the tumor’s morphological characteristics. Gorin et al. performed a multivariate analysis, including tumor characteristics, such as tumor diameter and hilar location, and reported that increased age, a larger tumor, and hilar location were significantly associated with pathologic upstaging [10]. Tay et al. performed a retrospective analysis of a very small cohort of 65 patients [20]. Even though they only analyzed a small number of patients, they included the R.E.N.A.L nephrometry score in their analysis and concluded that the tumor diameter, the central location within polar lines, and the total nephrometry score were significantly higher in patients with pathologic upstaging. However, they could not perform further multivariate analysis with other preoperative variables upon the occurrence of pathologic upstaging because of the small number of subjects.

Our study has certain limitations, including some inherent weaknesses due to its retrospective nature. We were unable to analyze the full spectrum of tumor morphologic features using the R.E.N.A.L nephrometry score. However, we attempted to evaluate the morphologic characteristics of pathologic upstaging in terms of hilar location and depth of the tumor. The follow-up period was not long enough to evaluate long-term survival outcomes. Furthermore, the patients with pathologic upstaging may have been more closely observed after surgery compared with patients without pathologic upstaging, which may have led to detection bias. We re-staged all the patients who initially underwent pathologic staging using the outdated TNM staging system prior to the introduction of the revised 2010 TNM classification. This process involved a thorough but retrospective central review of the initial pathologic reports, rather than actual re-inspection of all pathologic specimens, a major limitation of this study. In addition, detailed pathologic information about the cause of upstaging was not available. Finally, our study was only performed in Korean patients, and our results should be validated in other racial groups.

## Conclusion

There is a small but non-negligible incidence of pathologic upstaging from clinical stage T1 to pathologic stage T3a in patients with RCC. Factors such as patient age, tumor size, and hilar location are associated with upstaging. Patients with pathologic upstaging have a shorter survival than those without pathologic upstaging. However, partial nephrectomy does not compromise the oncologic outcomes in patients with clinical stage T1a RCC, even in the up-staged T3a patients. Thus, nephron sparing using partial nephrectomy should be considered in all patients diagnosed with small RCC.

## Abbreviations

BMI: Body mass index
CT: Computed tomography
RCC: Renal cell carcinoma
